# Cancer or Tuberculosis: A Comprehensive Review of the Clinical and Imaging Features in Diagnosis of the Confusing Mass

**DOI:** 10.3389/fonc.2021.644150

**Published:** 2021-04-28

**Authors:** Yufan Xiang, Chen Huang, Yan He, Qin Zhang

**Affiliations:** Department of Neurosurgery, Department of Oncology, Department of Postgraduate Students, West China School of Medicine, Sichuan University, Chengdu, China

**Keywords:** clinical diagnosis, cancer, tuberculosis, clinical feature, imaging feature, multimodal imaging, radiomics

## Abstract

Confusing masses constitute a challenging clinical problem for differentiating between cancer and tuberculosis diagnoses. This review summarizes the major theories designed to identify factors associated with misdiagnosis, such as imaging features, laboratory tests, and clinical characteristics. Then, the clinical experiences regarding the misdiagnosis of cancer and tuberculosis are summarized. Finally, the main diagnostic points and differential diagnostic criteria are explored, and the characteristics of multimodal imaging and radiomics are summarized.

## Introduction

Cancer and tuberculosis are two of the most common diseases affecting health worldwide. According to a recent World Health Organization (WHO) report, tuberculosis has one of the highest mortalities among all infectious diseases worldwide, causing an estimated 1.5 million deaths in 2018 (1). In 2020, an estimated 1,806,590 new cancer cases and 606,520 cancer-related deaths were estimated to occur in the USA. Tuberculosis is a great mimicker and diagnostic chameleon and is prone to be diagnosed as cancer (2). Moreover, due to its unusual presentations and lack of specific diagnostic tests, patients with cancer have been misdiagnosed with tuberculosis (3, 4). Therefore, these confusing masses represent a substantial clinical problem in the differential diagnosis of cancer and tuberculosis. Although many reports have addressed the differences between tuberculosis and cancer, comprehensive summaries are very rare (5).

Multimodal imaging involves the combination of at least two imaging tools to obtain more detailed, accurate images for diagnosis. Radiomics is a newly emerging form of computational medical imaging which involves the analysis and translation of medical images into quantitative data and have been rapidly developed in recent years. The analysis begins with acquiring a sufficient number of multimodal images of good quality and diversity. After extracting imaging features using a computer, trained algorithms can provide diagnostic aid or exact quantitative information by calculating the extracted features (6–9). Thus, the combination use of multimodal imaging and radiomics may enable a more accurate differential diagnosis for confused masses.

This review summarizes major theories designed to identify the factors associated with misdiagnosis and highlights key findings related to these confusing features to enhance diagnostic accuracy in clinical practice. Additionally, the potential for using multimodal imaging and radiomics in differentiating cancer and tuberculosis was also discussed.

## Methods

We searched PubMed for reported cases associated with the misdiagnosis of cancer and tuberculosis using the search strategy (“diagnostic errors” [Mesh]) AND (“tuberculosis” [Mesh])) AND (“neoplasms” [Mesh]) from 2000 to 2020. Among these patients, 11 of 37 were misdiagnosed with tuberculosis, and 26 of 37 were misdiagnosed with tuberculosis. Notably, these confusing diseases can involve any organ, such as the liver, salivary glands (10), kidneys (11), nasopharynx (12), pancreas (13), and gallbladder (14). The diagnoses of 33 of these patients were confirmed by biopsy of the lesion, while three were diagnosed based on their body fluid cultures, and one patient was diagnosed based on empiric antituberculosis treatment.

In summary, the clinical manifestations of these cases are non-specific. The main characteristics leading to the misdiagnosis of tuberculosis as cancer included false-positive positron emission tomography/computerized tomography (PET/CT) findings, an oncologic history and elevated carbohydrate antigen 125 (CA 125) or carbohydrate antigen 19-9 (CA 19-9) levels, while the main reasons for misdiagnosing cancer as tuberculosis were a history of tuberculosis, a positive tuberculin test, and a history of a rare cancer. The features associated with the chosen cases are summarized in Table 1.

**Table 1:** Feature summary of related cases.

**Study**	**Age**	**Gender**	**Misdiagnosis**	**Modified diagnosis**	**Clinical characters for leading misdiagnosis**
Hang et al. (2)	73	M	Hematogenous spread of gastrointestinal tumor	Atypical systemic hematogenous disseminated tuberculosis	CA19-9↑↑; CT findings of the two lungs showed multiple round or round-like nodules of different sizes, with clear boundaries and partial fusion
Di Renzo et al. (15)	76	M	Peritoneal carcinomatosis	Miliary tuberculosis	PET-CT resulting, thick, FDG-avid ring surrounding the liver
	53	M	Intrahepatic cholangiocarcinoma	Miliary tuberculosis	CT showed a large hypoattenuating mass and multiple prominent retroperitoneal, pericaval, and periportal lymph nodes; MRI showed a large liver lesion in the setting of cirrhosis; PET/CT demonstrated the right hepatic lobe mass to be FDG-avid
Muhammad et al. (10)	44	F	Salivary gland neoplasm	Salivary gland tuberculosis	Fine-needle aspiration cytology (FNAC) showed few atypical cells and the possibility of salivary gland neoplasm could not be ruled out
Li et al. (16)	71	M	Tuberculous pleural effusion	Pleural mesothelial sarcoma	Elevated adenosine dehydrogenase (ADH) and positive tuberculin test
Gandhi et al. (17)	3	F	Tuberculosis	Maxillary myxoma	Elevated ADA and normal tumor markers in pericardial effusion
Chaker et al. (11)	52	F	Renal cell carcinoma	Renal tuberculosis	History of pulmonary tuberculosis
Lee et al. (18)	37	M	Metastasis of breast carcinoma	Tuberculosis	History of breast cancer; PET/CT also showed intense uptake
	49	M			
	60	M			
Narahari et al. (19)	19	F	Tuberculosis	Invasive mucinous adenocarcinoma	Young patients
Kumawat et al. (20)	22	M	Tuberculosis	Chronic myeloid leukemia	Brain imaging and cerebrospinal fluid analysis suspected to have tubercular meningitis
Feng et al. (3)	45	M	Tubercular meningitis	Primary central nervous system lymphoma	MR images disclosed the swollen cerebellum and cauda equina, with contrast enhancement in both meninges and nerve roots and extremely high protein level in CSF
Arora et al. (21)	25	F	Squamous cell carcinoma	Primary oral tuberculosis	Oral ulcer with chronic non-healing history
Nyunt et al. (22)	39	F	Tuberculosis	Diffuse large B cell lymphoma	Chest X-ray showed an anterior mediastinal mass and computed tomography (CT)-guided biopsy was reported as chronic granulomatous inflammation suggestive of tuberculosis
Zhang et al. (23)	30	F	Nasopharyngeal carcinoma	Tuberculosis	Fibrolaryngoscope examination suggested nasopharyngeal carcinoma
Tembani (24)	24	M	Tuberculous pericardial effusion	Pericardial angiosarcoma	Large fibrinous pericardial effusions
Mou et al. (25)	65	F	Submucosal tumor	Esophageal tuberculosis	Endoscopy suggested submucosal tumor
Naselli et al. (25)	1	F	Mediastinal malignant neoplasia	Tuberculosis	A large mediastinal mass dislocating and compressing the respiratory structures
Liu et al. (26)	64	F	Tuberculosis	Intravascular large B cell lymphoma	CT showed left upper lobe pneumonia and tuberculosis skin test (PPD test) was positive
Yang (27)	40	M	Pancreatic carcinoma	Pancreatic tuberculosis	Computed tomography revealed a pancreatic mass that mimicked a pancreatic head carcinoma
Moghadam (28)	43	M	Gastric cancer	Gastric tuberculosis	The detection of negative acid-fast bacilli in the histopathology specimen
Zheng et al. (29)	45	M	Spinal tuberculosis	Spinal metastatic adenocarcinoma	CT/MRI of the lumbar spine supported the initial diagnosis of spinal tuberculosis
Agoda et al. (30)	27	M	Testicular cancer	Testicular tuberculosis	Ultrasonography suggested testicular cancer
Suárez et al. (13)	42	M	Pancreatic carcinoma	Pancreatic tuberculosis	CT revealed a heterogeneously enhancing, multicystic structure in the pancreatic head
Kim et al. (12)	72	M	Nasopharyngeal carcinoma	Nasopharyngeal tuberculosis	A potentially false-positive PET/CT finding
Basu et al. (31)	17	M	Neck recurrence in differentiated thyroid carcinoma	Tuberculosis	PET/CT found intense FDG uptake in the nodal conglomerate
Ringshausen et al. (32)	67	M	Metastatic lung cancer	Tuberculosis	Had no history of previous TB or TB exposure; negative result supported tuberculosis
Huang et al. (33)	44	F	Tuberculous spondylitis	Primary non-Hodgkin lymphoma	Primary non-Hodgkin lymphoma (PHL) of the spine is very rare
Bhatia et al. (34)	36	M	Branch-ducttype IPMT (BDT-IPMT), with liver metastasis	Pancreatic tuberculosis	Isolated pancreatic tuberculosis is a rare disease; CA19-9↑; The imaging features were suggestive of branch-ducttype IPMT (BDT-IPMT), with liver metastasis
Cantarella et al. (35)	52	F	Multifocal carcinoma	Glottic tuberculosis	Glottic tuberculosis is very rare
Ramia et al. (14)	64	M	Gallbladder cancer	Gallbladder tuberculosis	Gallbladder tuberculosis is very rare; false-positive PET/CT finding
Kong et al. (36)	74	M	Cerebral tuberculosis	Metastatic papillary adenocarcinoma	CT find a “target” lesion with a central core of calcification and a ring of enhancement.
Dursun et al. (37)	18	F	Endodermal sinus tumor	Peritoneal tuberculosis	Laboratory studies showed elevated CA125 and alpha fetoprotein levels suggesting an initial diagnosis of endodermal sinus tumor
Picolos et al. (38)	66	M	Metastasis of papillary thyroid carcinoma	Inactive pulmonary tuberculosis	History of thyroid cancer; positive result of radioiodine whole-body scintigraphy
Chen et al. (39)	80	F	Metastatic ovarian cancer	Pulmonary tuberculosis	Rapidly growing ovarian mass, elevated serum CA-125, and multiple pulmonary varying-sized nodular lesions
Gheorghe et al. (40)	63	F	Gastric cancer	Gastroduodenal tuberculosis	Gastroduodenal tuberculosis is a rare; endoscopy suggested gastric cancer
Kouraklis et al. (41)	35	F	Pancreatic carcinoma	Pancreatic tuberculosis	Frozen sections by direct trucut needle biopsy raised suspicions of a malignancy
O'Reilly et al. (42)	84	M	Breast carcinoma	Tuberculosis	Breast lump presenting clinically and radiologically as a carcinoma

*↑ and ↑↑, Increase*.

## Results and Discussion

### Imaging Modalities

#### Computerized Tomography (CT)

Computerized tomography (CT) is commonly used in clinical practice to initially assess whether a mass is a malignant tumor or benign nodule according to the imaging features such as the size, shape, tumor border, and enhancement characteristics. A previous study reported a misdiagnosis case based on a head and neck CT scan showing a target sign with a ring enhancement around a central nidus of calcification, which led to the misdiagnosis of metastatic papillary adenocarcinoma (originating from primary lung carcinoma) as cerebral tuberculosis (36). The target sign of cerebral tuberculosis was first described in 1979 (43) and became a pathognomonic requirement for a cerebral tuberculosis diagnosis in 1988 (44). As a non-specific radiologic finding, the target sign most commonly indicated cerebral tuberculoma or metastatic adenocarcinoma, and recent clinical evidence suggested its specificity for tuberculosis (45, 46). Cerebral tuberculosis also shows a solid enhancing mass (47). Therefore, in the different clinical contexts, this target sign confuses clinicians (36).

Chest CT scans lead to higher rates of misdiagnosis between tuberculosis and cancer than head and neck CT and abdominal CT. The CT scan features of pulmonary tuberculosis include irregular linear opacity, discrete miliary nodules, calcified nodules or consolidation, parenchymal bands, and pericicatricial emphysema (18). The most common findings of CT scans in patients with early bronchogenic spread of primary tuberculosis are 2–4 mm centrilobular nodules and branching linear lesions presenting as intrabronchiolar and peribronchiolar caseation necrosis. As the disease progresses, 2–4 mm centrilobular nodules may coalesce and become lobular and consolidated or expand to 5–8 mm nodules (48). After antituberculous chemotherapy, resolution of the lesion occurs, resulting in bronchovascular distortion, bronchiectasis, emphysema, and fibrosis (49). CT scans of early miliary dissemination commonly feature ground-glass opacification with barely discernible nodules, followed by discrete miliary nodules representing round intrapulmonary lesions of <3 cm (50). Multiple pulmonary nodular lesions of varying sizes usually lead to misdiagnosis, especially when complemented by non-specific symptoms (2, 26, 32). In some tuberculosis cases, chest CTs showed multiple round nodules of different sizes, with clear boundaries and partial fusion (2). In addition, chest CT scans have also shown pneumonia in patients with cancer. A previous study reported that chest CT scans of a patient with large B cell non-Hodgkin's lymphoma showed pneumonia throughout the entire lung and diffuse ground glass opacities in both lung fields (26). Furthermore, in patients with histories of tuberculosis and cancer, the imaging manifestations are so similar that definitive diagnoses are difficult to make, and errors occur more frequently than in patients without histories of these diseases (38, 39).

The common features of abdominal CT scans related to misdiagnosis are the pancreatic head (13, 27) and peritoneum (15). The incidence of pancreatic tuberculosis is rare, and this disease may present as a heterogeneously enhanced structure in the pancreatic head with multiple enlarged lymph nodes surrounding the head of the pancreas (13). These findings lead to errors in the diagnosis of patients suspected of having a pancreatic neoplasm with multiple lymph node metastases (27). Regarding miliary tuberculosis of the abdomen, abdominal CT scans may show a solitary liver mass with an irregular enhancing rim and progressive enhancement, which likely leads to a radiographic diagnosis of intrahepatic cholangiocarcinoma (15).

In general, the CT imaging features of spinal tuberculosis include irregular lytic lesions and sclerosis, narrowing of the intervertebral disk space, disc collapse with eventual progression to kyphotic deformity, destruction of the anterior parts of adjacent vertebrae, formation of a large paravertebral abscess, and calcifications or sequestra within the paravertebral abscess (51). The clinical characteristics of spinal tuberculosis and cancer metastasis are non-specific. The imaging presentations of spinal metastatic adenocarcinoma are highly consistent with spinal tuberculosis, and misdiagnosis occurs (29). On the other hand, radiotherapy is often used for suspected malignant spinal lesions without histologic confirmation, and a definitive diagnosis of spinal tuberculosis was finally made (52). Therefore, in cases of spinal lesions of unknown origin, tuberculosis should be taken into consideration despite a previous diagnosis of cancer.

#### Magnetic Resonance Imaging

Although the lung parenchyma was considered difficult to evaluate by magnetic resonance imaging (MRI) due to low proton density in the pulmonary tissue, susceptibility artifacts and respiratory motion artifacts (53), MRI would be helpful for discriminating pulmonary lesions because of its higher contrast resolution, absence of radiation, and multiple-parameter imaging (54). MRI has found relevant applications in the diagnosis of chest diseases and the differential diagnosis of benign and malignant lung lesions (55, 56). Qi et al. (57) investigated the differences in the imaging features of mass-like tuberculosis and lung cancer on conventional MR sequences and found that most tuberculosis lesions showed low signal intensity on T2-weighted images while lung cancer showed high signal intensity; the signal of tuberculosis lesions was mostly uneven on T2-weighted images, but the signal of lung cancer was mostly uniform; most tuberculosis lesions showed high signal intensity on T1-weighted images while lung cancer showed low signal intensity. Besides, benign mediastinal lymph nodes in tuberculosis lesions showed a variety of signals on T2-weighted images, whereas the majority of metastatic mediastinal lymph nodes displayed slight homogeneous hyperintensity.

#### Radiomics in Chest Imaging

Non-invasive and computer-aided alternatives have gradually been used in the differentiation of tuberculosis and lung cancer. In recent years, radiomics has attracted more and more attention due to its high-throughput extraction and distinguishing features from medical images, and to construct radiomics nomogram model to assist physicians to make the most accurate diagnosis (58–60). Cui et al. (61) developed and validated radiomics methods for distinguishing pulmonary tuberculosis from lung cancer based on CT images; they found the radiomics nomogram model exhibited good discrimination, with an AUC of 0.914 in the training cohort, and 0.900 in the validation cohort, showing that proposed radiomic methods can be used as a non-invasive tool for discrimination of tuberculosis and lung cancer on the basis of preoperative CT data. Another study (62) investigated the preoperative differential diagnostic performance of a radiomics nomogram in tuberculous granuloma and lung adenocarcinoma appearing as solitary pulmonary solid nodules and found that the radiomics nomogram showed better diagnostic accuracy than any single model with the AUC 0.9660, 0.9342, and 0.9064 for the training, internal validation, and external validation cohorts, respectively, which similarly indicated the radiomics nomogram could preoperatively distinguish between lung cancer and tuberculosis.

#### False-Positive PET/CT Findings

PET/CT is a powerful diagnostic method for characterizing masses, and it can more accurately assess mediastinal lymph nodes stages in cancer than CT (63). Many clinical conditions are now well-known to be responsible for false positives in oncological PET/CT scanning and are often related to uptake because of inflammation or infection processes. Infectious diseases, post-operative surgical conditions and radiation pneumonitis show as high fludeoxyglucose (FDG) uptake on PET/CT scans (64). Overexpression of glucose transporter-1 (GLUT-1) receptors in human macrophages, neutrophils, and lymphocytes following stimulation with cytokines or mutagens has been implicated in the intense uptake of FDG in inflammatory conditions. This highlights that standard uptake value (SUV) alone is an unreliable parameter for characterizing lesions in such a setting and should be used with caution and adequate correlation (31). A previous study showed that tuberculosis was mostly responsible (50%) for false positives in PET/CT (65). Tuberculosis at various locations, such as the gallbladder, nasopharynx, and peritoneum, is usually misdiagnosed due to false-positive findings on PET/CT scans (14, 15). In a case of nasopharyngeal tuberculosis, PET/CT revealed intense activity in soft tissue masses in the nasopharynx and cervical lymph nodes (12). Combined with those of fibrolaryngoscope examination, the results may lead to misdiagnosis as nasopharyngeal carcinoma (23). In lymph node tuberculosis, PET/CT also showed intense uptake. Patients previously diagnosed with cancer with multifocal hypermetabolic lesions may confuse clinicians, leading to misdiagnosis (66). Thus, when PET/CT findings show increased FDG uptake in patients with cancer as well as suspected metastatic lesions, tuberculous lymphadenopathy should be considered a differential diagnosis.

## Laboratory Tests

### Non-Specificity of CA 19-9 Levels in Serum

CA 19-9 is synthesized by pancreatic, gastric, colon, biliary ductal, and endometrial tissues, and its serum levels are extremely low (67). As a tumor marker for pancreatic, hepatobiliary, and gastrointestinal cancer, high CA 19-9 levels are indicative of advanced disease and a poor prognosis (CA 19-9 level of >100 U/ml usually suggests unresectable or metastatic disease) (68, 69). In addition to cancer, overexpression of CA 19-9 has also been observed in some benign conditions, including gastrointestinal disorders, hepatobiliary system diseases, pneumonia, pleural effusion, renal failure, and systemic lupus erythematosus (SLE), and this phenomenon may be associated with glycan-mediated cell-cell interactions in mucosal immunity (67). Hence, this indicator has low specificity. In the misdiagnosis of cancer and tuberculosis, sharply increased CA 19-9 levels also play a key role (2). CA 19-9 was reportedly elevated in cases of pulmonary tuberculosis (70), hematogenous disseminated tuberculosis (2), and pancreatic tuberculosis (34, 71). In pulmonary disease, the median CA 19-9 level in patients with pulmonary tuberculosis (median CA 19-9 level: 5.85 U/ml) was significantly lower than that in patients with pulmonary non-tuberculous mycobacterial disease (median CA 19-9 level: 13.80 U/ml, *p* < 0.001). Moreover, the CA 19-9 levels tended to decrease when pulmonary non-tuberculous mycobacterial disease was successfully treated, but this was not observed in pulmonary tuberculosis (70). Although the serum levels of CA 19-9 are commonly significantly lower in non-malignant diseases than in malignant diseases, CA 19-9 was reported to reach concentrations of 165 U/ml in hematogenous disseminated tuberculosis (2) and 66.84 U/ml in pancreatic tuberculosis (27). Therefore, although serum CA 19-9 levels lack specificity, CA 19-9 is the only marker of pancreatic cancer used in the clinic (69). When patients with increased CA 19-9 levels present atypically, tuberculosis may be the definitive diagnosis.

### Non-Specificity of Serum CA 125 Levels

Since the report on CA 125 expression in ovarian tumors published in the early 1980s, serum CA 125 levels have been utilized as a biomarker for the differential diagnosis of pelvic masses and have been widely used to monitor patients with ovarian cancer (72–74). Although the CA 125 levels in serum are elevated in over 80% of patients with ovarian cancer at the time of initial diagnosis, using this parameter as a diagnostic marker can lead to clinical mistakes (75). In regard to peritoneal tuberculosis, patients with rapidly growing ovarian masses and elevated serum CA 125 levels usually lead to an initial diagnosis of ovarian cancer (37). Furthermore, tuberculosis is misdiagnosed as a metastatic ovarian cancer when patients show elevated serum CA 125 levels and multiple pulmonary nodular lesions (39). Thus, infectious diseases can mimic metastatic diseases and therefore increase the difficulty of diagnosis.

### Adenosine Deaminase Activity

Adenosine deaminase activity (ADA) activity is widely distributed in human tissues and is the highest in lymphoid tissues, and two isozymes of ADA exist, ADA1 and ADA2 (76). The serum concentrations of ADA1 are high in patients with tuberculosis, and previous evidence suggests that the increased ADA1 is the results of T cell lymphocyte stimulation by mycobacterial antigens (77). ADA has been developed and widely used for the diagnosis of tuberculosis. However, it is not a specific index for differentiating between tuberculosis and cancer diagnoses. Although elevated ADA levels usually indicate tuberculosis, the non-specific characteristics of cancer patients presenting with increased ADA levels and a positive tuberculin test can lead to misdiagnosis (16, 17).

### Carcinoembryonic Antigen and Cytokeratin Fraction 21-1 in Serum

Both carcinoembryonic antigen (CEA) and cytokeratin fraction 21-1 (CYFRA 21-1) are widely used as tumor markers and are not considered to be confusing indexes based on the reviewed literature above. What is more, Jia et al. report that the CEA and CYFRA 21-1 levels are very valuable for distinguishing between lung cancer and pulmonary tuberculosis (78).

### Fine-Needle Aspiration Cytology

The diagnosis of cancer is usually made based on histological analyses of excisional biopsies, fine-needle aspiration cytology, and liquid biopsy (16). Fine-needle aspiration cytology, a commonly used method that is minimally invasive, quick, and accurate, involves the use of a thin needle to acquire cellular material from a bodily lesion or mass for diagnostic purposes. However, fine-needle aspiration cytometry is less sensitive and specific for some malignant tumors, such as those of the parotid gland (6), pancreas (41), and malignant lymphoma (79). In the parotid gland, the sensitivity and specificity of fine-needle aspiration cytology are 79 and 96%, respectively (80). In lymphoma, the median rate at which fine-needle aspiration cytology yields a subtype-specific diagnosis of lymphoma is 74% (77). Several factors are related to the specificity of fine-needle aspiration cytometry. First, fine-needle aspiration is unsatisfactory, and only the inflammatory necrosis area is obtainable in cases of small amounts of tissue, deep locations, and hard masses. Determining whether necrosis is caused by tumor disease or simple inflammation is almost impossible (81). Second, during pathological examination, seriously degenerated tumor cells are poorly stained, leading to fuzzy chromatin staining and structures. In particular, it is easy to make a misdiagnosis of tuberculosis when some carcinoma cells are spindle shaped, when nuclei exhibit vacuolar changes, and when lymphoma cells have lightly stained chromatin with obvious nucleoli and inflammatory necrosis (82). In addition, granulomatous inflammation often occurs in infectious diseases such as tuberculosis and mycosis and can also occur as a local inflammatory reaction in malignant tumors (83–85). Hence, although biopsy pathology is crucial for differentiating between cancer and tuberculosis, the findings of this examination may be a key factor underlying misdiagnosis.

## Other Characteristics

### History of Previous Disease

Tuberculosis and cancer have a complex and dangerous liaison relationship (86). On the one hand, long-term chemotherapy, radiotherapy, and surgery will weaken the immune system in patients with cancer, which increases the risk of tuberculosis infection (87, 88). On the other hand, a large cohort study reported that pulmonary tuberculosis is associated with an increased risk of developing lung cancers (89). In clinical practice, without specific information, patients that have been previously diagnosed with cancer or tuberculosis may be misdiagnosed (38, 66).

### Definitive Diagnoses of Rare Diseases

Physicians usually do not apt to consider diagnosis of patients as rare diseases at first time, such as primary non-Hodgkin lymphoma of the spine (33), glottic tuberculosis (35), isolated pancreatic tuberculosis (34), and gallbladder tuberculosis (14). The limited knowledge and preconceived professional ideas may lead to errors in the diagnoses of these rare cancers and tuberculosis.

## Conclusions

CT is commonly used in clinical practice to initially assess diseases. However, the shortcomings of this technology also cause confusion. Fine-needle aspiration cytology is a commonly used biopsy method but not reliable. Excisional biopsies may be the best definitive diagnostic method for confusing masses. When PET/CT findings show cancer patients with increased FDG uptake, tuberculosis should be considered a differential diagnosis, except in suspected cases of metastatic lesions. ADA and tumor markers are not specific indexes in the differential diagnosis of tuberculosis and metastasis. Thus, the following features are summarized to differentiate tuberculosis from cancer: (1) Fine-needle aspiration cytology has low sensitivity and specificity. (2) High FDG uptake on PET/CT scans can occur in cases of tuberculosis. (3) Tumor markers, including CA 19-9 and CA125, could be increased in tuberculosis patients. (4) ADA can be upregulated in in cancer patients. (5) Histopathological examination and tuberculosis cultures are still the gold standards for diagnosis.

In the diagnosis of tuberculosis, multimodal imaging could offer high spatial resolution, soft tissue contrast, and biological information at the molecular level with high sensitivity. Radiomics technology use computers to extract numerous imaging features, especially the ones that cannot be distinguished by eyes directly, which may enable the ability of automatically distinguishing complex images and offer quantitative information. However, the characteristics and quality of the images cannot be automatically assessed, which means the extracted features should be calculated by using a computational-aided diagnostic algorithms before they can be used practically (7, 90). With well-trained algorithms, confusing masses can be identified based on the extracted features. Beig et al. provided an excellent example of using radiomic features on lung CT images to distinguish adenocarcinomas from granulomas (8).

In summary, although clinicians should conduct more studies on the special features of these diseases, a comprehensive review of misdiagnosis characteristics could also guide the plan for use of multimodal imaging and radiomics-based algorithms.

## Author Contributions

QZ conceived and designed the experiments and revised the manuscript. YX, CH, and YH contributed reagents and materials and helped with the analysis. YX and CH wrote the manuscript. All authors contributed to the article and approved the submitted version.

## Conflict of Interest

The authors declare that the research was conducted in the absence of any commercial or financial relationships that could be construed as a potential conflict of interest.
